# Fat and Bone: An Odd Couple

**DOI:** 10.3389/fendo.2015.00190

**Published:** 2016-03-07

**Authors:** Richard Kremer, Vicente Gilsanz

**Affiliations:** ^1^McGill University, Montreal, QC, Canada; ^2^Children’s Hospital Los Angeles, Keck School of Medicine of the University of Southern California, Los Angeles, CA, USA

**Keywords:** bone, fat, imaging, vitamin D, marrow fat, MRI, hormones

## Abstract

In this review, we will first discuss the concept of bone strength and introduce how fat at different locations, including the bone marrow, directly or indirectly regulates bone turnover. We will then review the current literature supporting the mechanistic relationship between marrow fat and bone and our understanding of the relationship between body fat, body weight, and bone with emphasis on its hormonal regulation. Finally, we will briefly discuss the importance and challenges of accurately measuring the fat compartments using non-invasive methods. This review highlights the complex relationship between fat and bone and how these new concepts will impact our diagnostic and therapeutic approaches in the very near future.

## Introduction

We will briefly review how the definition of osteoporosis has evolved to integrate other parameters in addition to bone mineral density (BMD) measurements. We will then review the makeup of the bone microenvironment and the distribution of fat within and outside the bone compartment. Finally, we will briefly summarize how muscle and its fat composition may have impact on bone strength.

### The Concept of Bone Strength

At the National Institutes of Health (NIH) Consensus Conference in 2000, osteoporosis was defined as a skeletal disorder characterized by compromised bone strength that predisposes to an increased risk of fracture (1). Bone strength reflects the integration of two features: BMD and bone quality. BMD is one of the strongest risk factor for fractures and its measurement has long been used to define osteoporosis. Clinical risk factors have also been integrated with BMD measurements in an attempt to help clinicians better identify patients requiring osteoporosis therapy (2). The FRAX calculator is a user-friendly web-based tool that provides immediate quantification of risks and treatment decision making based on a very simple algorithm. However, it should always be interpreted within the clinical context as it does not take into account a number of important clinical variables.

The World Health Organization defines osteoporosis as two and a half SD below the peak bone mass [i.e., the maximum amount acquired post bone maturation around the age of 18 in women (3) and 20 in men (4), but bone growth can continue up to the age of 30]. It is expressed as grams of mineral per area or volume. On the other hand, bone quality reflects a combination of bone microarchitecture, bone turnover, and mineralization. Peak bone mass is therefore a critical parameter that will impact bone strength as the skeleton is aging. Genetic factors appear to account for over 50% of the variation in peak bone mass acquisition (5). As bone is progressively lost overtime, the higher the peak bone mass, the longer the skeleton could theoretically withstand damage. This progressive bone loss from peak bone mass occurs predominantly as a result of reduced bone formation from osteoblast (6, 7) and resultant protein composition (8) and persists for decades thereafter. Additionally, accelerated bone resorption predominates in women as a result of estrogen deficiency at the menopause but also to a lesser extent in men after the fifth decade (6). However, postmenopausal women have the ability to produce estrogens from the peripheral conversion in fat tissues of testosterone to estradiol. Adipocytes indeed express the cytochrome P450 enzyme, aromatase, which can produce estradiol from testosterone. This peripheral production of estradiol has been proposed as a protective mechanism against bone loss in overweight women (9–11).

Bone strength is highly dependent on its structural and material properties. The balance between bone formation and resorption, also called bone turnover, greatly influences the material properties of bone such as tissue mineral density and collagen cross-linking. Enhanced bone turnover, as seen with a lack of estrogen in postmenopausal women, influences the structural and material properties that lead to bone microdamage. With aging, the reduction in bone strength is further compounded by progressive muscle weakness and the increased risk for falls due to lack of balance and coordination. Maintenance of bone mineralization within a relatively narrow range is also critical to the maintenance of bone strength (12). Poorly mineralized bone loses its stiffness, whereas excessive mineralization makes bone more brittle. Bisphosphonates, the most widely used drugs to treat osteoporosis by excessive bone turnover, also lead to increased mineralization and stiffness overtime. Impairment of microdamage repair is another potential side effect of bisphosphonates since normal bone turnover replaces old bone with new bone and protects against microdamage. Long-term use of bisphosphonates has been linked to atypical fractures, and one could hypothesize that the combination of abnormal mineralization and reduced turnover may play a role in its development.

### The Components of the Bone Microenvironment

The bone microenvironment is comprised of several compartments, including hematopoietic cells, bone cells, and stromal cells (13). Bone cells, also referred to as the bone remodeling unit (BMU), are composed of bone-forming osteoblasts, bone-resorbing osteoclasts, and osteocytes embedded within the bone matrix. The BMU is also in close contact to stromal elements of the marrow and the blood vessels supply (14). Osteoclasts are of hematopoietic origin, whereas osteoblasts originate from bone marrow mesenchymal stem cells (MSCs) (11, 14, 15). One of the most interesting occurrences in this environment is the accumulation of fat cells during aging and in some pathological conditions. The functional significance of this “marrow fat (MF)” accumulation correlates strongly and inversely with bone strength (16). However, its causal relationship to bone degradation as well as its potential for therapeutic targeting in osteoporosis remains to be determined. There is indeed a significant gap of knowledge in our understanding of the mechanistic relationships between fat and bone especially during the aging process.

### The Components and Distribution of Body Fat

In humans, white adipose tissue (WAT) is principally located beneath the skin (subcutaneous fat) and around internal organs (visceral fat or abdominal fat). The main cellular component of WAT is the adipocyte but other cell types are also present, including fibroblasts, macrophages, and blood vessels. Its main function is energy storage. Adipose tissue also accounts for a significant proportion of the breast tissue and is found around other organs (such as pericardial and gonadal fat) providing protective padding. Adipocytes are also found in small amounts outside adipose tissues, including muscle, liver, pancreas, and heart, which are also referred as ectopic fat. Fat cells are also found in the bone marrow, “MF,” and have been the subject of enormous research interest to explore their relationship with the bone microenvironment.

Another form of adipose tissue is known as brown fat or brown adipose tissue (BAT) located mainly around the neck and large blood vessels of the thorax of neonates whose main function is to generate heat and protect neonates against cold (17). Recent studies indicate that BAT is also found in the neck and trunk of adults albeit in lesser amounts (18). Although this review focuses mainly on white fat, the relationship between BAT and bone will be briefly discussed.

### Muscle Fat, Muscle Strength, and Bone Strength

Many studies have clearly demonstrated the positive impact of muscle strength on bone strength, but we will not cover this important area of research here. However, an interesting, but much less explored, area is the relationship between muscle fat and muscle strength and by extension its impact on bone strength. Fat accumulation in muscle may also have indirect effects on bone. Intermuscular adipose tissue accumulation occurs during aging or in pathological conditions such as Duchenne muscular dystrophy, which has been linked to decreased muscle strength, a known risk factor for osteoporosis and fractures (19). Increased intermuscular adipose tissue is associated with poor mobility (20) and increased risk of hip fractures (21). However, it is not yet known whether intermuscular adipose tissue accumulation is simply a marker of muscle dysfunction or has a direct causal effect on muscle function. The relationship between vitamin D and intermuscular adipose tissue is discussed later in this review.

## Basic Understanding of the Mechanistic Relationship Between Marrow Fat and Bone Strength

In this section, the origin, clinical significance, and the factors that influence MF accumulation will be discussed.

### Clinical Observations

As we age, the cortex of the bones become thinner encircling concomitantly larger marrow cavities filled with fat, but whether this is a result of a passive accumulation of fat as bone is lost and marrow space increases or an age-related shift in MSC differentiation with predominant adipogenesis against osteoblastogenesis is difficult to elucidate.

Meunier et al. studied 81 iliac crest biopsies from elderly women and found that bone marrow samples from women with osteoporosis had a pronounced accumulation of adipocytes, relative to levels in healthy young subjects (22). Subsequent studies showed increased bone marrow adiposity in postmenopausal women with osteoporosis and a negative association between bone–MF and rate of bone formation (23–25). Investigations using magnetic resonance imaging (MRI) have shown that the accumulation of bone–MF in the vertebral bodies of older women with low bone mass confers an additional risk for compression fracture beyond that associated with low BMD (26). Further support for this notion are data showing an association between exogenous glucocorticoid use and endogenous over production of cortisol and marked bone marrow infiltration by adipocytes with a significant increase in fracture risk (27–29).

Marrow stromal cells isolated from postmenopausal osteoporotic patients express more adipocytic differentiation markers than those with normal bone mass and are more likely to enter an adipocyte than an osteoblast differentiation program (30, 31). Fat in bone marrow may also promote bone resorption since marrow adipocytes, much like fat cells elsewhere, secrete inflammatory cytokines capable of recruiting osteoclasts (32).

### Role of MSC

Pluripotent bone marrow MSCs have the ability to become osteoblasts, chondrocytes, myocytes, or adipocytes under the influence of specific cell-derived differentiation factors (33). This process has been well demonstrated *in vitro* to control the fate of MSC into osteoblasts or adipocytes. This process is bidirectional and considerable plasticity has been observed both *in vitro* and *in vivo* in the ability of bone cells to become adipocytes and *vice versa*. Mechanical stimuli on the skeleton can also modify the differentiation of MSC into the cell lineages responsible for bone and fat formation (34–39) such that increases in bone strain add to increased osteogenic activity, whereas decreases favor the adipogenic differentiation. Lastly, the lack of estrogen in rats following oophorectomy has been reported to lead to profound fatty bone marrow infiltration, suggesting that estrogen must play an important role in regulating adipocyte recruitment (40).

**Figure 1:**
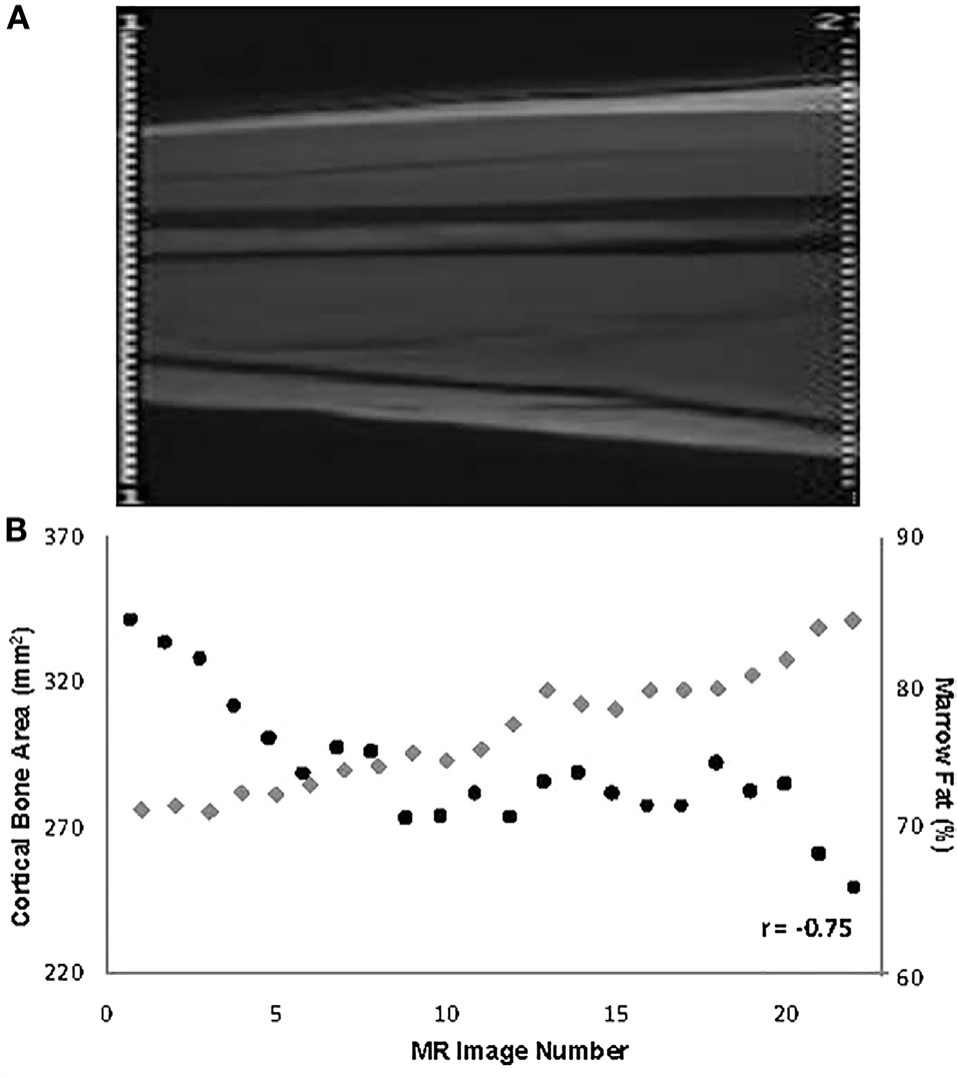
**(A)** Depiction of the mid-third of the right femur in a 19-year-old male (the localizer image). **(B)** Values for % marrow fat (black circles) and cortical bone area (gray diamonds) at all slices along the mid-third of the right femoral shaft and their overall relationship in the same subject [reproduced from Wren et al. (46)].

Data from pathological specimens and imaging studies have consistently observed a reciprocal relationship between bone mass and increased marrow adiposity in elderly humans (22, 41–43). A recent study found that the accumulation of MF during aging is linked to increased expression of RANKL, a finding that could explain at least in part age-related bone loss (44). However, whether the relation between these two tissues in the elderly represents the clinical translation of preferential differentiation by MSC into the adipose cell lineage or is merely the unintended consequence of a passive accumulation of adipose tissue as bone is lost and marrow space increases has been a matter of considerable debate. To avoid this confounding effect, young subjects were examined and found that bone acquisition is tightly linked with decreases in marrow adiposity (16). The inverse relation between the amount of bone and MF is observed at all sites along the shaft of the bone in the young and the old regardless of age, gender, or anthropometric measures (45, 46) (Figure 1). Moreover, prospective longitudinal studies have found that bone acquisition in the appendicular skeleton of healthy young females is inversely related to changes in marrow adiposity (16). Consequently, one could make a strong argument that during the aging process, differentiation of MSCs into adipocytes is favored at the expense of osteoblasts, resulting in MF accumulation and decreased bone mass. However, this causal relationship has not yet been demonstrated.

In summary, mounting evidence supports a mechanistic relationship between MF accumulation and bone loss, pointing out the potential to target this pathway to prevent or even reverse the process of bone aging.

## Basic Understanding of the Relationship Between Body Fat, Body Weight, and Bone Strength

In this section, we will summarize the current knowledge and conflicting data linking body fat, bone mass, and fracture rate.

### Clinical Observations

Postmenopausal women have the ability to produce estrogens from the peripheral conversion of testosterone to estradiol in fat tissues. Adipocytes express the cytochrome P450 enzyme, aromatase, which can produce estradiol from testosterone. This peripheral production of estradiol has been proposed as protective mechanism against bone loss in overweight women (9–11). There are also reports showing an inverse relationship between BMI and osteoclast activity in normal postmenopausal women (47) and an increase in bone resorption following weight loss (48). As discussed further in the next section, fat accumulation leads to hyperinsulinemia, which is anabolic to bone, and adipocytes produce estrogen and adiponectin, which have a positive effect on bone strength and could therefore explain this positive association observed clinically (49).

Bone mineral density measured by dual-energy X-ray absorptiometry (DXA) is positively related to body weight and BMI (49, 50), possibly because higher body weight may increase mechanical loading on the skeleton, a mechanism known to stimulate bone formation. However, DXA measurements are falsely elevated by increased body fat and therefore DXA may overestimate BMD in obese individuals (51–54). Indeed, other studies have found a strong positive association between lean mass and BMD in young women and a much weaker association between BMD and fat mass (50).

A meta-analysis indicates that a high BMI appears to protect against fractures at any site in both men and women (55). Similarly, a European study found that a higher BMI protects against vertebral fractures (56). In the study of osteoporotic fractures, body weight in the lowest quartile was found to double the risk of hip fracture (57). In contrast, other studies found that the risk of hip fractures is positively correlated with fat mass in a cohort of French women (58) and Chinese men (59). Interestingly, visceral adiposity has been linked to deterioration of bone structure and skeletal fragility (60, 61), suggesting that fat compartments may have different effects on bone strength. In summary, clinical observations linking body fat and bone strength are inconsistent, and more mechanistic studies are needed to support the purported beneficial effect of obesity on osteoporosis.

## Integrated Hormonal Regulation of Fat and Bone

In this section, we will review the major hormonal regulators controlling fat and bone, with particular attention on the mechanisms underlying the reciprocal relationship between MF and bone.

### Growth Factors

#### Insulin

Hyperinsulinemia is a hallmark of the metabolic syndrome characterized by accumulation of visceral fat (62). Osteoblasts express insulin receptors (63), and insulin directly stimulates osteoblast proliferation (64) and differentiation *in vitro* (63). Furthermore, local application of insulin over the calvariae of adult male mice produces a significant increase of bone formation (65). Conversely, the glucose-dependent insulinotropic polypeptide (GIP)-receptor knockout mouse shows decreased bone size, mass, and formation rate (66). In clinical studies in patients with varying degrees of hyperinsulinemia, the risk of vertebral fracture was inversely related to insulin levels (67). Hyperinsulinemia following an oral glucose load is accompanied by suppression of parathyroid hormone (PTH) production and bone turnover and may therefore indirectly protect against bone loss (68, 69).

#### Growth Hormone and Insulin-Like Growth Factor 1

*In vitro* GH induces MSC differentiation into osteoblast, while GH deficiency in mice results in decreased bone formation and increased bone marrow adiposity (70). Although IGF-1 does not have a direct effect on the differentiation of MSC *in vitro* (71), the PPAR-gamma 2 agonist rosiglitazone decreases IGF-1 expression in bone marrow MSC and lowers blood IGF-1 levels in mice and humans (72).

#### GLP-1 and GLP-2

Administration of glucagon-like peptide-1 (GLP-1) to diabetic mice results in an insulin-independent anabolic effect on bone (73). In humans, dietary fat and protein leads to reduction in bone turnover (74–76) possibly through GLP glucagon-like peptide-2 (GLP-2), a polypeptide produced by intestinal L cells in response to feeding. GLP-2 administration to humans is accompanied by a reduction in bone resorption and an increase in bone density (55).

### Adipokines

#### Leptin

Leptin is primarily produced by adipocytes and initially discovered as an appetite suppressant (77). The hypothalamus is regarded as the principal target of leptin. The arcuate nucleus (in the hypothalamus) contains anabolic neurons, which express both neuropeptide Y and agouti-related protein, the activity of which is inhibited by leptin, and neurons expressing pro-opiomelanocortin (POMC), which are activated by leptin. Insulin acts on both types of neurons in the same way as leptin, suggesting that these hormones reinforce each other’s actions centrally, as well as peripherally (78).

However, subsequent studies demonstrated the potent effect of leptin on bone in animal studies (79, 80). These studies demonstrated that in obese mice deficient in leptin (ob/ob mice) or in mice where the leptin receptor is defective (db/db mice), vertebral trabecular bone volume and bone formation are increased. Conversely, intracerebroventricular infusion of leptin decreased vertebral trabecular bone volume and bone formation (79). Further studies then demonstrated that these effects are mediated by the sympathetic nervous system acting on β-adrenergic receptors at the surface of osteoblasts inhibiting bone formation (80). These inhibitory effects on bone *in vivo* contrast with the *in vitro* effects reporting that leptin directly promotes the differentiation of osteoblasts (81–85). Leptin also reduces expression of RANK ligand of human bone marrow stromal cells and RANK expression of peripheral blood mononuclear cells (81, 86), with a resultant inhibition of osteoclastogenesis (82, 86). Furthermore, clinical studies have not consistently showed a relationship between administration of beta blockers, bone density improvement, and fracture prevention (87). This apparent contradiction could be explained by the ability of leptin to act positively and directly on peripheral tissues or negatively *via* central mechanisms involving activation of the sympathetic nervous system.

#### Adiponectin

Adiponectin is another adipokine produced by adipocytes whose role is to increase insulin sensitivity. Its blood levels are decreased in obese and diabetic individuals (88, 89). *In vitro* treatment of osteoblasts with adiponectin enhances their differentiation (90). In humans, cross-sectional studies found an inverse association between circulating adiponectin levels and bone mass in both men and women, even after adjustment for fat mass (91, 92).

### Peroxisome Proliferator-Activated Receptor (PPAR) Gamma

PPARγ2 is the most important regulator of adipogenesis. *In vitro* PPARγ2 directs the commitment of MSCs into adipocytes and inhibits their differentiation into osteoblasts (9). Ablation of the PPARγ gene leads to enhanced osteoblastogenesis of embryonic stem cells *in vitro* and results in enhanced bone mass *in vivo* and reduced bone marrow adiposity (93). At the cellular level, *ex vivo* examination of MSCs shows commitment toward osteoblastogenesis and reduced adipogenesis (94). Administration of rosiglitazone, a specific activator of PPARγ, in mice decreases osteoblastogenesis and enhances adipogenesis in the bone marrow (95).

The canonical Wnt/beta-catenin pathway and non-canonical Wnt signaling have been implicated in this reciprocal regulation *via* PPAR-gamma 2. In the canonical pathway following ligand activation, Wnt binds to a transmembrane coreceptor complex consisting of Frizzled receptors and LRP5 to stimulate bone formation (96). Although Wnt10b, Wnt 3a, and Wnt 7 can stimulate the differentiation of MSC into osteoblast while inhibiting adipogenesis (97–101), only Wnt 7 has been shown to block PPAR-gamma 2 (99). Similarly, the non-canonical ligand Wnt5a was found to induce Runx2-mediated osteoblastogenesis while simultaneously suppressing adipogenesis in bone marrow MSC through the formation of a corepressor that blocks PPAR-gamma 2 gene transcription (102). In addition, PPAR-gamma 2 acts downstream of the Wnt receptor complex to enhance the proteosomic degradation of beta catenin, thereby acting as a direct regulator of osteoblastogenesis (103).

### Cytokines

Duque et al. provided *in vitro* and *in vivo* evidence that interferon (IFN)-gamma is a potent inducer of MSC differentiation into mature osteoblasts and a key regulator of bone formation in mice and has therefore the potential to become an efficient drug target in osteoporosis (104, 105). It was further demonstrated that IFN-gamma inhibits adipogenesis *in vitro* and prevents MF infiltration in oophorectomized mice *in vivo* (106). In addition, IFN-gamma suppresses osteoclast differentiation by interfering with RANKL signaling (107), thus acting synergistically on bone cells to enhance bone strength. As discussed earlier and independently of IFN-gamma, MF accumulation during aging is linked to increased expression of RANKL, highlighting another mechanism linking bone loss to MF (44). It remains to be established whether other proinflammatory and anti-inflammatory cytokines could affect the balance between MF and osteoblastogenesis.

### Glucocorticoids

Excessive production or supre-physiological administration of GC excess results in inhibition of osteoblastogenesis and accelerated adipogenesis (108) through suppression of Wnt signaling (109) and induction of PPAR-gamma 2 expression (110).

### Calcium-Regulating Hormones

#### Vitamin D

Vitamin D insufficiency is a worldwide phenomenon affecting even the sunniest areas (111–113).

Vitamin D (from skin irradiation or in the diet) must be metabolically activated first by the liver 25 hydroxylase (CYP2R1) to 25hydroxyvitamin D (25OHD) and then by the kidney 1αhydroxylase to its active form 1,25dihydroxyvitamin D [1,25(OH)_2_D]. The role of vitamin D on bone and mineral homeostasis is well known, but its role in other tissue function including fat is still the subject of considerable debate.

The relationship between vitamin D and fat has been the subject of many studies in recent years. Several studies found a strong and inverse correlation between circulation levels of 25OHD and weight but also BMI (113–117) in both men and women across the ages. Furthermore, this inverse association was seen in all fat compartments but was stronger for visceral fat (113), perhaps indicative of higher cardiovascular morbidity. In support of this, several studies showed that decreased 25OHD levels impair insulin action (118–120) and are associated with the metabolic syndrome (120–122). At the cellular level, mechanistic studies also support this association. 1,25(OH)_2_D treatment of pre-adipocytes in culture decreases adipogenesis (123) through inhibition of C/EBPalpha and PPARγ;VDR and PPARγ act synergistically to inhibit adipogenesis (124). The effect of vitamin D on MF has also been examined in animal studies. Our group showed that continuous administration of 1,25(OH)_2_D in senescence-accelerated mice (SAM-P/6) suppressed adipogenesis in the marrow and that isolated MSCs had a reduced expression of the adipogenic enhancer PPARγ (125) and accelerated differentiation into osteoblasts compared to placebo-treated animals. This was accompanied by an increase in both cortical and trabecular bone strength (126). Other studies also support our data that vitamin D enhances MSC differentiation to osteoblasts (127, 128), suggesting that 1,25(OH)_2_D may exert a protective effect on bone aging.

However, a causal relationship supporting the role of vitamin D as a regulator of fat metabolism and distribution in humans has been difficult to prove. In support of this theory, Ortega et al. found that baseline 25OHD levels are predictive of the efficacy of weight loss regimen and that the vitamin D status potentiates the effect of low caloric diet (129). Several other studies showed that vitamin D supplementation induces a moderate effect on weight loss while others did not (130–135). On the other hand, other clinical studies point to the evidence of fat as a reservoir for vitamin D (sequestration theory). First, it was shown that obesity is directly related to 25OHD levels: sequestration hypothesis (136, 137) and second, that weight loss tends to increase 25OHD levels (138).

An unexplored effect of vitamin D action on bone could come from its effect on fat accumulation in muscle. Vitamin D is also a major determinant of skeletal muscle function (139–142). A severe lack of vitamin D can cause myopathy (143, 144), which tends to be more marked in the proximal muscles (145). In the elderly, vitamin D deficiency is linked to muscle weakness and increased susceptibility to falls and fractures, which improve with administration of vitamin D with calcium (146–157). We recently found that in healthy young women, vitamin D levels are inversely related to the degree of fat infiltration in muscle (112), a phenotype associated with impaired muscle strength (19, 20, 158). Available data indicate higher muscle lipid content to also be associated with decreased muscle function in patients with neuromuscular disorders (19). Indeed, even in healthy subjects, higher muscle lipid content is associated with lower levels of muscle strength and physical performance, independent of muscle mass (20, 158). Among the different mechanisms that could explain the accumulation of fat in muscle, it is tempting to speculate that mesenchymal progenitors normally present in skeletal muscle – MSCs, muscle-derived stem cells, or muscle satellite cells – could potentially allow muscular growth and regeneration or differentiate into cells with an adipocyte phenotype, including the abilities to express adipocyte-specific genes and accumulate lipids (159–161).

**Figure 2:**
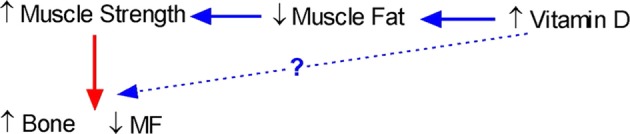
**Conceptual model of the interrelationship between bone, fat, and muscle and the role of vitamin D**. An increase in vitamin D should lead to a decrease in muscle fat leading to an increase in muscle strength and a subsequent increase in bone and simultaneous decrease in marrow fat (MF). However, there is also the possibility that increases in vitamin D will also directly lead to an increase in bone.

Our studies showing that vitamin D is inversely related to fat infiltration in muscle (112) and positively related to muscle strength (139) in healthy young females support the notion that vitamin D may be a key determinant of muscle precursor cell (MPC) differentiation. However, whether vitamin D-mediated muscle adiposity and performance determine bone acquisition, and simultaneous decreases in marrow adiposity remains to be determined. A conceptual relationship between muscle, bone, and fat and how it could be influenced by vitamin D is shown in Figure 2.

Most vitamin D supplementation trials on muscle strength have been done in the elderly and found a reduction in falls, improvements in balance and body sway, and/or resolution of myalgia in statin-treated patients with treatment periods as short as 8–12 weeks (146, 162, 163). Likewise, several studies have examined the impact of vitamin D supplementation on muscle composition, primarily by assessing muscle fiber number and diameter, infiltration of fat and fibrosis, and all were in elderly subjects (152, 164, 165); treatment with vitamin D and calcium improved muscle composition after as short a time as 3 months. The ability to obtain tissue samples from healthy, young women undergoing surgery for sports-related injuries represents a unique approach in this field of research.

Two vitamin D supplementation studies have been done in girls; one found improvements in muscle function in the vitamin D-treated group but no significant differences in bone measures using DXA and peripheral quantitative computed tomography (CT), while the other found increases in DXA measures of lean mass and spine bone mineral content (139, 166). These discrepant results likely reflect the limitations of the techniques employed. Changes in body composition influence DXA measures during growth, and peripheral quantitative CT measures in children have poor reproducibility due to large variations in bone growth (167, 168). Confounding effects associated with growth and development are common when studying sexually and skeletally immature young women, using DXA or CT.

### Parathyroid Hormone

Parathyroid hormone is a major regulator of calcium and bone homeostasis, but studies on its effect of fat have been so far limited. Two epidemiological studies suggest a possible positive association between circulating levels of PTH and fat mass. The first showed that circulating PTH concentrations are directly correlated with fat mass (169), and the other showed that body weight is increased in women with primary hyperparathyroidism as compared to controls (170).

## Brown Adipose Tissue and Bone

Much of this review focused on the interaction between white fat and bone, which is by far the most studied. In contrast, the literature on BAT and bone is almost non-existent except for two correlative studies showing that a positive relationship exists between BAT and bone volume in children and adolescent boys and girls (171), and BAT and bone size in both children and adults (171, 172). The effect of BAT became insignificant when muscle mass was introduced in the model, a finding supported by a previous study showing a positive relationship between BAT and muscle mass (173). It has also been reported that young women with active BAT have higher BMD than women without BAT (174), further supporting a possible mechanistic relationship between BAT, bone growth, and bone strength. The underlying mechanism(s) remains to be established.

## The Challenges of Fat Imaging

In this section, we will briefly summarize the recent progress in non-invasive measurement of MF and body using imaging technologies.

Studies assessing MF–bone interactions have been hindered by the difficulty of independently examining different tissues at the same site. The most commonly employed method to assess bone and body composition has been DXA, which cannot analyze muscle or MF. In contrast, CT and MRI provide accurate measures of bone, muscle, and fat independently (175–179). MRI has the added advantage of being able to quantify the amount of any tissue without exposing the subject to radiation. However, MRI measurements of bone, bone–MF, and muscle require state-of-the-art imaging, including Dixon capabilities. Over the past 25 years, Dixon’s method has evolved significantly (19, 177, 180–183), and recent advances have led to more generalized algorithms (176, 184–186). We used three-point Dixon MR technique for fat quantification (182) and determined that reproducibility of the fat fraction quantifications in phantom models was excellent with a coefficient of variation of <1.5% (182). *In vivo* reproducibility of MF varies between 1.3 and 3% (176, 177). Pixel signal intensities from the medullary canal are obtained, and total fat % is calculated by integration in slice selection direction over the imaging volume. To calculate bone structural properties from the MRI images, our group has developed a graphical user interface with Matlab (Mathworks, Natick, MA, USA) using custom algorithms. The program is designed to automatically extract endosteal and periosteal contours of the bone and to calculate geometric and structural parameters. First, the user selects a DICOM image and then crops a rectangular region of interest containing the bone of interest. The image is automatically thresholded according to bone and muscle peaks from the image histogram. Edges of the cortex are detected and contours generated. The correlation of this method with quantitative CT is excellent (187). Multiple investigators have previously evaluated properties of the femoral midshaft using tracing (188), deformable models (189), and semi-automatic algorithms.

Possible differences in the distribution of fat accumulation in children have been difficult to establish due to the limitations and the risks of the techniques used. While there are many techniques, including underwater weighing, anthropometry, body water dilution, impedance, and DXA, to estimate total body fat content, it has not been possible to differentiate between subcutaneous and visceral fat until the advent of CT and MRI (190). Both CT and MRI provide a three-dimensional assessment of body tissues (175). CT provides cross-sectional images from which the amounts and distributions of subcutaneous fat and visceral fat are well distinguished, but these determinations are areal measurements (cm^2^) and multiple scans are necessary to obtain true volumetric values, exposing the child to radiation. In contrast, MRI allows for volume determinations and can reliably measure the amount and distribution of abdominal fat, without radiation exposure (cm^3^) (175, 179). In a study comparing MRI with five other methods (underwater weighing, O dilution, K counting, skinfold thickness, and body electrical impedance methods), MR gave the least variability and an estimate of body fat significantly closer to the mean of the five other methods than any other technique alone. MRI-based studies are likely to be less affected by individual variability and may therefore achieve higher statistical power for a given sample size (178).

## Conclusion

The interactions between fat and bone are complex and new emerging concepts regarding their relationship have the potential of transforming our therapeutic targeting of the skeleton. The inverse relationship between MF and bone is an enthralling area of research based on the very origin of bone and fat cell differentiation from MSC. The obesity epidemic has also brought new challenges in terms of prevention and treatment of common illnesses, such as type 2 diabetes. Here again, the interactions between body weight, body fat, and bone are much more complex, and the influence of clinical context, age, sex, and ethnicity should be considered when examining this relationship. Overall, bone and fat may not be such an odd couple but rather a very important one that deserves to be examined in all its facets as it represents a unique challenge for future health.

## Conflict of Interest Statement

The authors declare that the research was conducted in the absence of any commercial or financial relationships that could be construed as a potential conflict of interest.
